# Puerarin Alleviates Vascular Cognitive Impairment in Vascular Dementia Rats

**DOI:** 10.3389/fnbeh.2021.717008

**Published:** 2021-10-15

**Authors:** Tiantian Zhu, Moli Zhu, Yue Qiu, Zeqing Wu, Ning Huang, Guangrui Wan, Jian Xu, Ping Song, Shuangxi Wang, Yaling Yin, Peng Li

**Affiliations:** ^1^College of Pharmacy, Xinxiang Medical University, Xinxiang, China; ^2^Henan International Joint Laboratory of Cardiovascular Remodeling and Drug Intervention, Xinxiang, China; ^3^Xinxiang Key Laboratory of Vascular Remodeling Intervention and Molecular Targeted Therapy Drug Development, Xinxiang, China; ^4^School of Basic Medical Sciences, Xinxiang Medical University, Xinxiang, China

**Keywords:** vascular dementia, puerarin, ROS, TRPM2, NR2A, cognitive impairment (dementia)

## Abstract

Cerebral ischemia triggers vascular dementia (VD), which is characterized by memory loss, cognitive deficits, and vascular injury in the brain. Puerarin (Pur) represents the major isoflavone glycoside of Radix Puerariae, with verified neuroprotective activity and cardiovascular protective effects. However, whether Pur ameliorates cognitive impairment and vascular injury in rats with permanent occlusion of bilateral common carotid arteries (BCCAO) remains unknown. This work aimed to assess Pur’s effects on BCCAO-induced VD and to dissect the underlying mechanisms, especially examining the function of transient receptor potential melastatin-related 2 (TRPM2) in alleviating cognitive deficits and vascular injuries. Rats with BCCAO developed VD. Pur (50, 100, and 150 mg/kg) dose-dependently attenuated the pathological changes, increased synaptic structural plasticity in the dorsal CA1 hippocampal region and decreased oxidative stress, which eventually reduced cognitive impairment and vascular injury in BCCAO rats. Notably, Pur-improved neuronal cell loss, synaptic structural plasticity, and endothelial vasorelaxation function might be mediated by the reactive oxygen species (ROS)-dependent TRPM2/NMDAR pathway, evidenced by decreased levels of ROS, malondialdehyde (MDA), Bax, Bax/Bcl2, and TRPM2, and increased levels of superoxide dismutase (SOD), Bcl2, and NR2A. In conclusion, Pur has therapeutic potential for VD, alleviating neuronal cell apoptosis and vascular injury, which may be related to the ROS-dependent TRPM2/NMDAR pathway.

## Introduction

Vascular dementia (VD) represents a commonly diagnosed dementia in elderly individuals, only following Alzheimer’s disease (AD) in terms of prevalence among dementia types (O’Brien and Thomas, 2015). VD, characterized by memory loss, cognitive deficits, and vascular injury in the brain (Kalaria et al., 2016), is mostly attributed to stroke whose risk factors show independent associations with elevated risk of vascular cognitive impairment (Sahathevan et al., 2012). Mouse nerve growth factor (NGF) is the most important neurotrophic and regenerative drug independently developed in China. It is used for the survival and functional maintenance of central cholinergic neurons and widely used in ischemic hypoxic encephalopathy, sequelae of stroke, craniocerebral injury, and other neurological diseases (Xu and Wang, 2016; Lu and Chen, 2017; Zhang et al., 2019a). However, No FDA-approved drug is currently available for VD, which has many etiologies still requiring further investigation (Korczyn et al., 2012). Most VD patients show cognitive impairment, partly due to hypoperfusion-related ischemia, oxidative stress, and neuroinflammation in the brain, which all have associations with VD (Bar et al., 2003). It is anticipated that the number of stroke survivors with cognitive impairment will remarkably rise in the near future, particularly in the elderly (Dichgans and Leys, 2017). In general, novel targeted anti-VD drugs with higher efficacy are urgently needed (Liu et al., 2018).

Oxidative stress, reflected by elevated reactive oxygen species (ROS) amounts, represents an important characteristic of VD (Zhang J. et al., 2015; Mai et al., 2020). Puerarin (Pur), or 7-hydroxy-3-(4-hydroxyphenyl)-1-benzopyran-4-one 8-(β-D-glucopyranoside), constitutes the major isoflavone glycoside produced in *Pueraria lobata’s* root (Jiang et al., 2005). It has been utilized for multiple medical conditions in traditional Chinese medicine for a long time (Qicheng, 1980). Pur has been shown to be neuroprotective in different neurological pathologies such as AD, Parkinson’s disease, brain ischemia and BCCAO-induced VD (Lin et al., 2012; Zhang et al., 2012; Zhu et al., 2014; Zhang J. et al., 2015). Pur also exerts effects on vascular dysfunction (Wei et al., 2014). However, the mechanisms by which oxidative stress induces BCCAO-associated brain and vascular damage remain unclear, as well as how Pur exerts neuroprotective and vascular protective effects.

Transient receptor potential melastatin-related 2 (TRPM2), an ion channel, modulates ROS-associated cell death in multiple cells (Jiang et al., 2010; Jiang and Mortadza, 2018). In neurons, the GluN2A-containing *N*-methyl-Daspartate (NMDA)-mediated signaling pathways are neuroprotective, via which TRPM2 modulates ischemia-reperfusion induced brain “delayed neuronal death” (Alim et al., 2013; Ye et al., 2014) that commonly referred to pyramidal neurons in hippocampal CA1 region are especially vulnerable and these neurons are demised after transient ischemia in rodents, non-human primates and humans (Pulsinelli et al., 1982; Ye et al., 2014). Moreover, the pathological mechanism of nerve cell damage is also accompanied by calcium overload (Lin et al., 2007; Xu and Zheng, 2007), which is associated with TRPM2 (Belrose and Jackson, 2018) and the NMDA subfamily of glutamate receptors (NMDAR) (Vieira et al., 2020). Thus, Pur may exert neuroprotective effects though ROS-dependent TRPM2/NMDAR pathway in VD Rats.

Therefore, we hypothesized that Pur may prevent BCCAO-induced excessive production of ROS, protecting cerebral neurons and endothelium-dependent vasorelaxation from oxidative stress-induced cell injury by interrupting the TRPM2/NMDAR pathway.

## Materials and Methods

### Drugs and Animals

Puerarin (Pur, >98% HPLC purity) was provided by Aladdin (Shanghai, China). Male Wistar rats (180–200 g) were obtained from SPF (Beijing) Biotechnology Co., Ltd. All animal experiments followed the National Institutes of Health guide for the care and use of Laboratory animals (NIH Publications No. 8023, revised 1978). This study involving animals had approval from the Xinxiang Medical University Veterinary Medicine Animal Care and Use Committee.

### Animal Experiments

The common carotid arteries of rats, which underwent adaptive housing for 7 days and were fasted for 12 h, were submitted to bilateral and permanent occlusion by ligation (Ji et al., 2010). The operation was performed under inhalation anesthesia (5% isoflurane + 95% oxygen for induction; 3% isoflurane + 97% oxygen for maintenance). A ventral midline incision exposed internal and external carotid arteries that were delicately separated from the vagus nerve. Then, the bilateral common carotid arteries were double ligated using 5-0 silk sutures with no ligation in sham operated animals. Penicillin powder in adequate amounts was sprinkled on the wound before careful closure.

**FIGURE 1:**
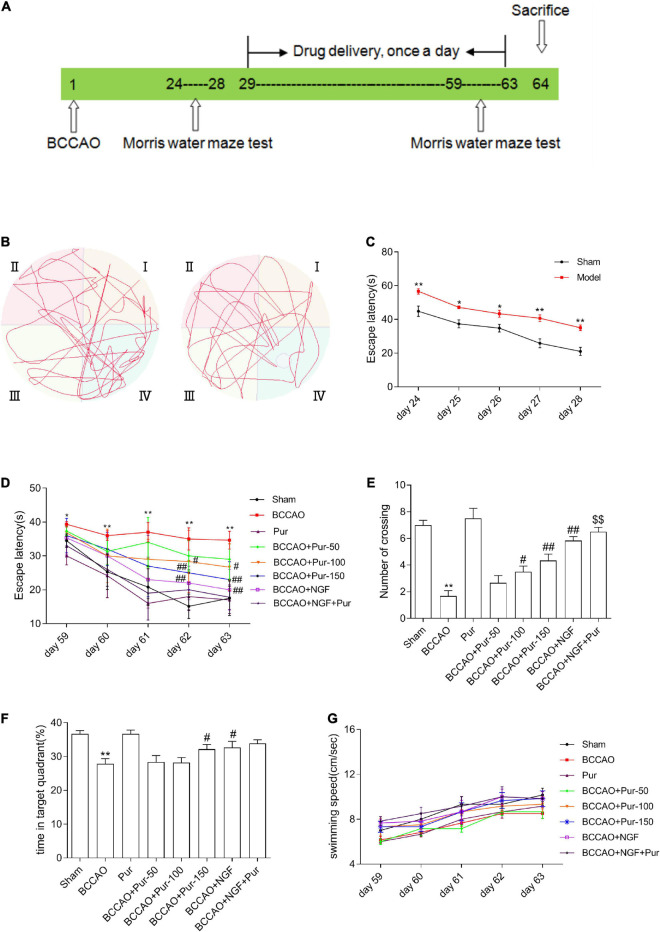
Effects of puerarin (Pur) on occlusion of bilateral common carotid arteries (BCCAO)-induced spatial memory impairment. **(A)** Timeline of surgery (BCCAO), drug administration and behavioral assays. **(B)** Results of the Morris water maze (MWM) (days 28 following BCCAO) in the sham and BACCO groups. **(C)** Escape latency (starting site to the hidden platform) at days 24–28 post-BCCAO). **(D)** Escape latency at days 59–63, during Pur treatment. **(E)** Numbers of crossings in the target quadrant within 60 s in the probe trial on day 63. **(F)** Percentages of time spent in the target quadrant within 60 s in the probe trial (no platform) on day 63. **(G)** Swimming speed (path length by escape latency) in each group at days 59–63. Values were mean ± SEM (*n* = 6). *P < 0.05, **P < 0.01 vs. Sham; ^#^*P* < 0.05, ^##^*P* < 0.01 vs. BCCAO; ^$$^*P* < 0.01 vs. BCCAO + Pur (150 mg/kg/day).

The Morris water maze (MWM) was performed at 24–28 and 59–63 days after operation in behavioral experiments. The MWM was performed at 24–28 days to exclude unsuccessfully modeled rats. The mean time required for reaching the hidden platform by the rat in the acquisition phase on five consecutive days was determine. In our training protocol, we allowed mice to locate the platform within 1 min and if unsuccessful, they were visually guided to the platform in order to match the experience of the “learners.” This was typically done in two ways. The first was by putting a finger on the platform, so the goal became more salient. If the mouse still failed to swim to the platform, we placed the hand in the water directly in front of the mouse to guide it there. This training might be required on initial acquisition trials and could be terminated as soon as a mouse located the platform in a single trial before maximum time expires. If failures persisted, the mouse was tested for improved performance the next day. However, if it failed again, it is excluded from the experiment (Pan et al., 2020). The screening criterion (SC) was according to the mean time of each BCCAO rat and the mean time of all sham rats, and the formula was as follows: SC = BCCAO-sham/BCCAO. A rat whose SC was greater than 0.2 was considered to have a cognitive deficit” (Zhao et al., 2002; Liu et al., 2018). Next, the animals were assigned to eight groups (*n* = 6), including the sham (sham-operated rats administered 0.9% saline), sham plus Pur [sham-operated rats administered high-dosage Pur intragastrically (i.g.) at 150 mg/kg/day], BCCAO (BCCAO rats treated with 0.9% saline for five continuous weeks), BCCAO plus Pur (BCCAO animals treated with Pur i.g. at 50, 100, or 150 mg/kg/day for 5 weeks), BCCAO plus mouse NGF (BCCAO rats were administered NGF i.g. at 20 mg/kg/day for 5 weeks), and BCCAO plus Pur and NGF [BCCAO rats administered Pur (150 mg/kg/day) and NGF (20 mg/kg/day) i.g. for 5 weeks] groups. Pur (50, 100, or 150 mg/kg), NGF (20 mg/kg) and vehicle (0.9% saline) were administrated i.g., from day 29 post-operation to day 63, respectively. The MWM was performed at 59–63 postoperative days for evaluating Pur’s effects in improving cognitive impairment in rats. Behavioral assays were carried out 4 h upon treatment with Pur. The detailed procedure for animal experiments is shown in Figure 1A. Sodium pentobarbital (30 mg/kg, i.p.) was employed for anesthesia, and euthanasia occurred on day 64.

Freshly isolated hippocampal samples (*n* = 6 per group) were divided into three portions. One portion was snap frozen and kept at –80°C for subsequent protein expression analysis. The second was utilized for immunofluorescence, terminal deoxynucleotidyl transferase dUTP nick end labeling (TUNEL) and Golgi-Cox staining, and the remainder portion of the hippocampus underwent 4% paraformaldehyde fixation and stained with hematoxylin and eosin (H&E).

### The Morris Water Maze Test

As mentioned above, the MWM is used for behavioral assessment of learning and memory (He et al., 2012). The MWM system consists of a heated swimming pool (120 cm in diameter and 70 cm in height) with obvious pictures on four directions of the pool wall, a movable rat platform (12 cm in diameter and 30 cm in height), a video recording system (fixed 100 cm above the heated swimming pool to record the movement of the rats), a computer and analysis software. The MWM was carried out in the heated swimming pool, which was divided into four quadrants. In the experiment, a movable rat platform was placed in the third quadrant, the water surface was about 0.5 cm above the platform, water temperature was maintained at 22 ± 0.5°C, the environment was kept quiet and the experimenters could not been seen. During the test, the time and trajectory used by the rats which were entered into water via the ?quadrant for locating the platform were recorded at the same time by the video recording system above the pool. The experiment had two phases, including the training stage (the first to the fourth day during MWM test) and a probe test stages (the fifth day during MWM test). For each training trial, each rat entered the water from different quadrants each day for training to find the platform. The latency to escape onto the hidden platform and the path length were recorded. The rat was given a maximum of 60 s to find the hidden platform. If the rat failed to find the platform within 60 s, the training was terminated and a maximum score of 60 s was assigned. The rat was then guided to the hidden platform, and it was allowed 10 s reinforcement on the platform. In the second stage of the MWM test, after the last training trial on training day 4, a probe test, in which the hidden platform was removed, was conducted the next day. The rat was allowed to swim freely for 60 s in the pool before it was removed from water, and the number of times it crossed the platform and the mean time spent in the target quadrant were recorded by the computer tracking system (Columbus Instrument) connected to the camera as an assessment of spatial memory (Li et al., 2018; Liu et al., 2018).

### Vascular Ring Test

Cervical dislocation with subsequent decapitation was utilized for euthanasia. Middle brain arteries were exposed, dissected, and placed in chilled aerated Krebs-Henseleit solution (NaCl, 119 mM; KCl, 4.7 mM; NaHCO_3_, 25 mM; MgSO_4_, 1.0 mM; glucose, 11.1 mM; KH_2_PO_4_, 1.2 mM; and CaCl_2_, 2.5 mM). This was followed by connective tissue removal. The obtained middle brain arteries were sliced in 1.5–2 mm wide rings, of which one underwent mounting in myograph-containing Krebs-Henseleit solution (pH 7.4) at 37°C and bubbling with 95% O_2_ and 5% CO_2_. A force transducer coupled to the Powerlab data acquisition system (Ad-Instruments, Australia) was utilized to record isometric contractions. Middle brain artery rings were primed with 80 mM KCl for functional integrity assessment and contractility improvement. Cumulative dose responses for ACh (10^–8^–10^–4^ M) and sodium nitroprusside (SNP; 10^–8^–10^–4^ M) were obtained in phenylephrine (3 × 10^–6^ M)-precontracted preparations with untouched and denuded endothelia, respectively (Verma and Sharma, 2015).

### Neuropathological Analysis

For hematoxylin and eosin (H&E) staining, paraffin blocks, embedded with a paraffin embedding machine (HistoCore Arcadia H, Leica microscopic system Co., Ltd.; batch number: 14039357259), were sectioned at 5 μm using a paraffin slicer (batch number: 14050237960, LEICA RM2255; Leica Microsystems Co., Ltd.). After xylene dewaxing, gradient ethanol dehydration (70–100%), H&E staining and xylene treatment, the sections were sealed with neutral gum and observed under an optical microscope.

The TUNEL assay (Beyotime Biotechnology, China, C1088) was carried out to detect cell apoptosis, as directed by the manufacturer and described in a previous report (Wang et al., 2015). Hippocampal tissues were fixed and embedded in OCT to prepare frozen sections. Coronal slices of 6 μm were prepared by the frozen slicing mechanism, and baked at 37°C for 30 min. After baking, the tissue slice was fixed with 4% formalin (30–60 min) and submitted to two PBS washes of 10 min each. This was followed by a 5-min incubation with 0.5% Triton X-100 in PBS at room temperature and two PBS washes. Totally 50 μl of TUNEL detection solution was added to each sample for 1 h at 37°C. Upon 3 PBS washes, anti-fluorescence quenching solution was utilized for sealing the sections, which were analyzed by fluorescence microscopy (Olympus, Tokyo, Japan).

Golgi-Cox staining (Servicebio, China, G1069) was carried out to detect the morphologic changes of synaptic structural plasticity, as directed by the manufacturer (Mariotto et al., 2018). Hippocampal tissues were fixed and embedded in OCT to prepare frozen sections. Coronal slices of 80 μm were prepared with the frozen slicing mechanism and dried at 37°C for 4 h. Then, the slices were placed in 5% potassium dichromate solution for 3 days, rinsed with double distilled water 2–3 times, and incubated with 2% silver nitrate solution at 37°C for 3 days. Finally, 95–100% ethanol was used to dehydrate the samples, followed by xylene treatment and sealing with neutral gum. The slices were observed and photographed by oil immersion microscopy (Olympus, Tokyo, Japan). The density of dendritic spines was calculated according to the previous researches (Zhang, 2018; Pan et al., 2020). Three dendrites from each of five different nerve cells in the dorsal CA1 hippocampal region of the rats were analyzed. Dendritic spines were contrast-adjusted with the ImageJ software (National Institutes of Health, Bethesda, MD, United States) to allow for greater accuracy in their counting. The numbers of mushroom and filopodia spines were counted and divided by the total length of the dendrite. The results have been expressed as the number of spines identified per 100 μm of dendrite.

### Immunofluorescence

Upon OCT embedding, the obtained hippocampal tissues were sliced and incubated at ambient. After 3 PBS washes of 5 min each, 0.5% Triton X-100 was utilized for permeabilization for 10 min, followed by 3 PBS washes as above. Goat serum was employed for blocking. Then, the samples were successively incubated with primary antibodies targeting Bax (1:200, Affinity, #AF0120), Bcl2 (1:200, Affinity, #BF9103), TRPM2 (1:200, Affinity, #DF7533), and NR2A (1:100, Abcam, ab240884), 4°C overnight, respectively, and secondary fluorescent antibodies (Servicebio, China, 1:100, GB22301 or 1:300, GB21303), 2 h at ambient. The LeicaTCSSP2 laser scanning confocal microscope (Leica, Germany) was employed for imaging and analysis.

### Reactive Oxygen Species ELISA

Mitochondrial superoxide levels were assessed with the MitoSOX Red Kit (Invitrogen, United States) (Zhang et al., 2019b). In brief, 20 μg of isolated mitochondria was added to 50 μl of respiration buffer in 96-well plates. Then, 100 μl of chilled respiration buffer containing 2 μM MitoSOX, 5 μM antimycin A and 4 μM ADP were added. Finally, 50 μM salermide or 1 μM FeTCCP was supplemented to the indicated wells, followed by fluorescence reading every 5 min (background at 355 and 590 nm).

### SOD and MDA ELISA

Approximately 2 ml of blood collected with heparin tube (KG051NH) was extracted from the common carotid artery. The extracted blood was placed into a tube to mix well. The supernatant was obtained by centrifugation at a speed of 3,000 rpm (4°C, 15 min). Following the manufacturer’s instructions of an ELISA kit (Sigma, United States), blood samples were detected for related oxidative indexes.

### Immunoblot

Protein extraction from bilateral hippocampal tissue specimens was carried out with radioimmunoprecipitation (RIPA) buffer (150 mM NaCl, 50 mM Tris–HCl pH = 7.4, 10 mM EDTA pH = 8.0, 1% Triton X-100, 1% deoxycholate, and 0.1% SDS, in PBS) containing 0.1% phenylmethylsulfonyl fluoride (PMSF). Total protein amounts were determined in lysates cleared by centrifugation. Equal amounts of total protein (20–60 μg) were resolved by 10% sodium dodecyl sulfate polyacrylamide gel electrophoresis (SDS/PAGE) and electro-transferred onto polyvinylidene fluoride (PVDF) membranes (Millipore, United States). Upon blocking (5% skim milk) at ambient for 1 h, membranes underwent successive incubations with primary (4°C overnight) and peroxidase-linked mouse anti-rabbit (1:5,000; Santa Cruz, United States; ambient for 1 h) antibodies. Primary antibodies targeted Bax (1:500; Affinity, #AF0120), Bcl2 (1:1,000; Affinity, #BF9103), TRPM2 (1:1,000; Affinity, #DF7533), NR2A (1:1,000; Abcam, ab240884) and β-actin (1:5,000; Affinity, AF7015). Easy See Western Blot Kit (Beijing TransGen Biotech, China) was utilized for development. Data analysis by densitometry was performed with Image J software (National Institutes of Health, Bethesda, MD, United States).

### Statistical Analysis

Data analysis was performed with SPSS 18. Data were expressed as mean ± SEM. Statistical analysis was performed by two-way mixed ANOVA for MWM test escape latencies and swimming speeds or one-way between subjects ANOVA for the other data. The differences between two groups was tested by Fisher’s least-significant difference *post hoc* test. *P* < 0.05 indicated statistical significance.

## Results

### Puerarin Attenuates Spatial Cognitive Impairment Associated With Occlusion of Bilateral Common Carotid Arteries

Occlusion of bilateral common carotid arteries is the classical modeling methods of VD (Liu et al., 2018; Li et al., 2020). During our modeling phase (1–28 days), the survival rate of BCCAO rats was 64.71% (24/68). Learning and memory retention in the MWM were utilized for spatial memory evaluation in rats (Liu et al., 2018). During the identified time (24–28 days), the rate of successfully modeled rats (Supplementary Table 1) that had cognitive deficits was 81.82% (8/44). Then, we used successfully modeled rats to conduct the follow-up experiments.

As depicted in Figures 1B,C, escape latency (time to locate the hidden platform) was reduced at 24–28 days in the totality of animals. However, BCCAO rats showed prolonged escape latency during training compared with sham animals. The data of swim distance, swim time, and swim speed were shown in Supplementary Figure 1. And all these data showed that hypoperfusion effectively caused learning deficit in the BCCAO model. After Pur (100, 150 mg/kg) and NGF (20 mg/kg) treatment for 5 weeks, escape latency was markedly shortened in comparison with the BCCAO group on days 4–5 (Figure 1D). Retention performance was examined as described above. As depicted in Figure 1E, BCCAO rats had fewer crossings than other groups. Pur (100 or 150 mg/kg) or NGF (20 mg/kg) treatment improved this deficit on day 59. Moreover, Pur (100 or 150 mg/kg) plus NGF (20 mg/kg) treatment had a better effect than Pur (150 mg/kg) treatment alone. In Figure 1F, BCCAO rats showed reduced mean time spent in the target quadrant in comparison with all other groups. Pur (150 mg/kg) and NGF (20 mg/kg) treatment improved this deficit on day 59. To assess whether group differences in escape latency resulted from distinct swimming abilities, swimming speed (path length/escape latency) was determined, and values were similar among groups (Figure 1G).

### Puerarin’s Effects on Middle Brain Artery Relaxation and Histological Features

**FIGURE 2:**
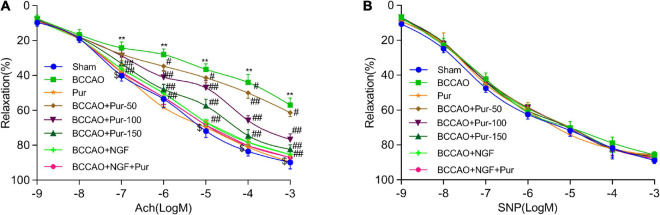
Effects of Pur on middle brain artery relaxation. **(A)** Pur’s effect on acetylcholine-associated endothelium-dependent vasorelaxation in BCCAO rats. **(B)** Pur’s effect on sodium nitroprusside-associated endothelium-independent vasorelaxation in BCCAO rats. Values were mean ± SEM (*n* = 6). **P < 0.01 vs. Sham; ^#^*P* < 0.05, ^##^*P* < 0.001 vs. BCCAO group; ^$^*P* < 0.05 vs. BCCAO + Pur (150 mg/kg/day).

Endothelial independent vasodilation refers to the relaxation of smooth muscle cells directly caused by drug or physiological stimulation. However, endothelium-dependent relaxation refers to the relaxation of smooth muscle cells depending on the function of endothelial cells. Under drug or physiological stimulation, endothelial cells could convert l-arginine (L-Arg) to endothelium-dependent relaxation factor NO via nitric oxide synthase (NOS), then NO causes the relaxation of smooth muscle cells through cCMP pathway (Rees et al., 1989). Acetylcholine and sodium nitroprusside dose-dependently cause endothelium-dependent and -independent relaxations, respectively (He et al., 2012). BCCAO markedly alleviated acetylcholine-associated endothelium-dependent relaxation. Pur (50, 100, and 150 mg/kg/day, i.g.) and NGF (20 mg/kg/day, i.g.) remarkably improved acetylcholine-associated endothelium-dependent relaxation in BCCAO animals. However, SNP-associated endothelium-independent vasorelaxation was not remarkably affected. The results shown in Figure 2A indicated that BCCAO impaired endothelium-dependent relaxation in the rat middle brain artery. Treatment with Pur or NGF, specially Pur and NGF together, increased maximum relaxation to ACh in BCCAO rats without affecting ACh-related relaxation in normal animals. Finally, Pur or NGF had no effect on SNP-induced relaxation in all rats (Figure 2B).

### Effects of Puerarin on Neuropathological Changes Associated With Occlusion of Bilateral Common Carotid Arteries

**FIGURE 3:**
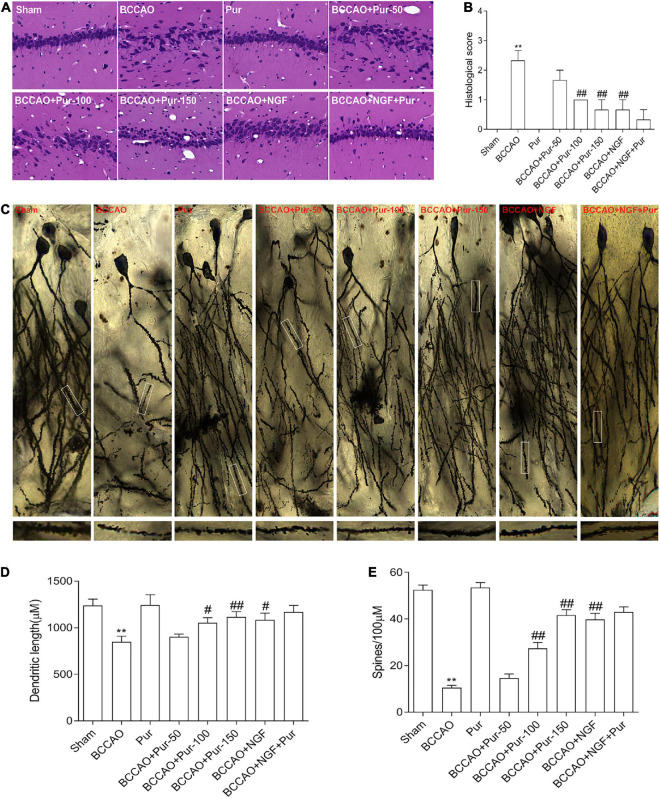
Pur’s effects on morphological alterations and dendrites in the hippocampus of BCCAO rats (H&E staining and Golgi-Cox staining). **(A)** HE staining in groups, Representative micrographs are shown at ×200. **(B)** Histological score. **(C)** Golgi staining in groups. **(D)** Percentage of mushroom spines. **(E)** Number of spines. Values were mean ± SEM (*n* = 6). **P < 0.01 vs. Sham; ^#^*P* < 0.05, ^##^*P* < 0.001 vs. BCCAO group.

The dorsal CA1 region of hippocampus is critical in learning and memory, and very susceptible to ischemia (Vago et al., 2007). Death of hippocampal neurons represents an important factor affecting memory impairment (Choi et al., 2014). Figure 3A depicts representative micrographs of H&E staining of hippocampus specimens 64 days post-BCCAO. Overt neuronal alterations were observed, including multiple nerve cells arranged sparsely and disorderly, unclear boundary between the cell contour and surrounding tissue and neuronal cell loss. Treatment with Pur or NGF starkly alleviated the above pathological alterations as well as BACCO-related cell loss in the dorsal CA1 region of hippocampus. The histological score was shown in Figure 3B. Golgi staining detects the synaptic structure in the hippocampal CA1 region (Pan et al., 2020). As demonstrated in Figure 3C, neurons atrophied, axon branches decreased, fractured, and exhibited bead-like structures, and the number of spines and percentage of mushroom spines (Figures 3D,E) decreased in BCCAO rats. After Pur or NGF administration, The synaptic structure was restored to a certain extent. Moreover, Pur treatment dose-dependently recovered the synaptic structure.

### Puerarin’s Effects on Neuron Apoptosis Induced by Occlusion of Bilateral Common Carotid Arteries

**FIGURE 4:**
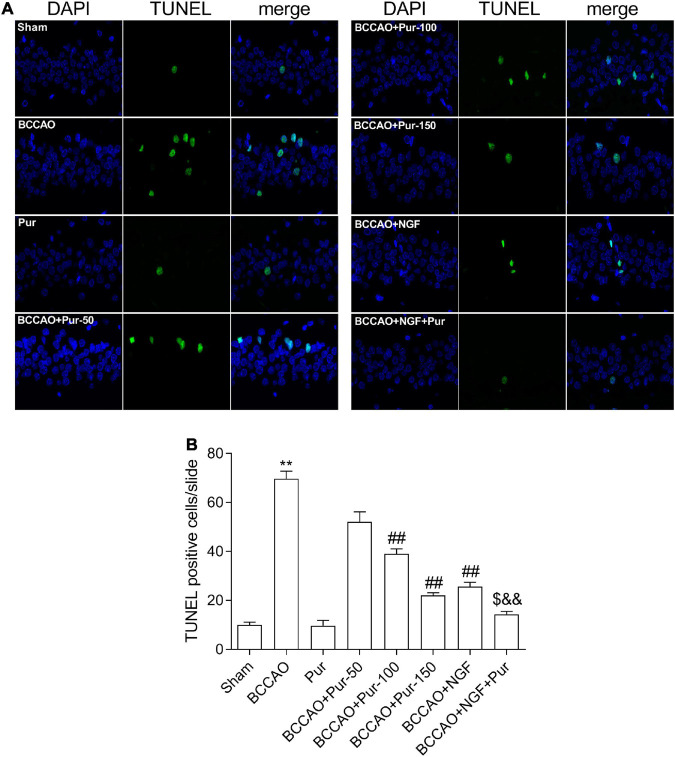
Puerarin’s effects on apoptosis in dorsal CA1 region of hippocampal neurons in BCCAO rats (TUNEL staining). **(A)** Tunel staining to identify positive apoptotic cells (Red arrows), representative micrographs are shown at ×40. **(B)** The statistics of TUNEL positive cells. Values were mean ± SEM (*n* = 6). **P < 0.01 vs. Sham; ^##^*P* < 0.01 vs. BCCAO; ^$^*P* < 0.05 vs. BCCAO + Pur (150 mg/kg/day); ^&&^*P* < 0.01 vs. BCCAO + NGF (20 mg/kg/day).

**FIGURE 5:**
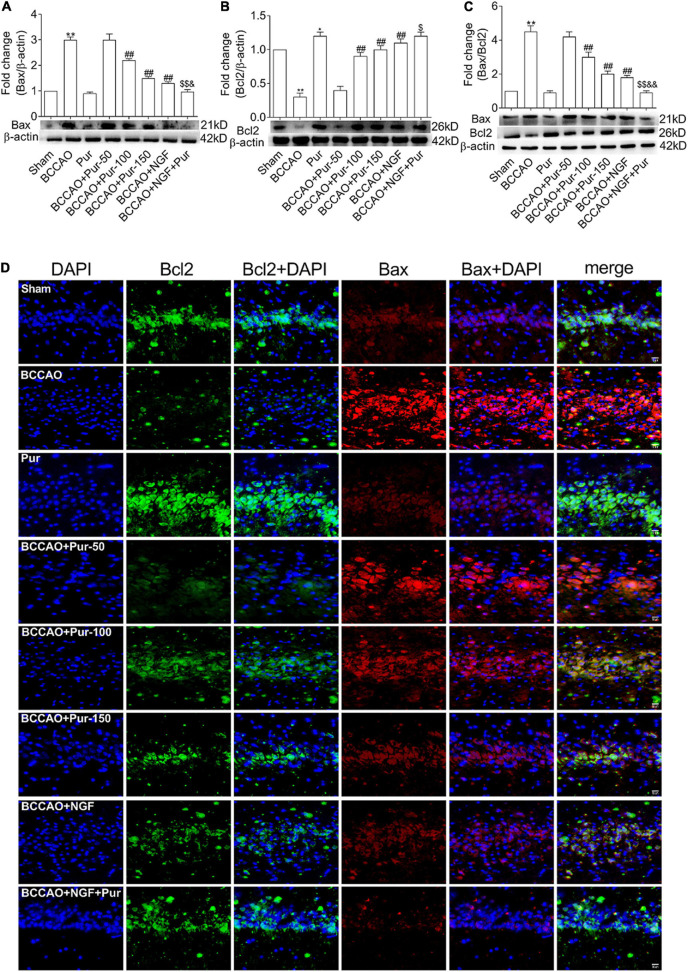
Expression and co-localization of Bax and Bcl2 in the rat dorsal CA1 region of hippocampus. **(A)** Protein expression of Bax in the hippocampal dorsal CA1 region of rats. **(B)** Protein expression of Bcl2 in the hippocampal dorsal CA1 region of rats. **(C)** Bax/Bcl2 ratios in the hippocampal dorsal CA1 region of rats. **(D)** Pur’s effects on Bax and Bcl2 co-localization in the hippocampal dorsal CA1 region of rats, assessed by immunofluorescent double staining. Representative micrographs are shown at ×200. Results were mean ± SEM (*n* = 6). *P < 0.05, **P < 0.01 vs. Sham; ^##^*P* < 0.01 vs. BCCAO; ^$^*P* < 0.05, ^$$^*P* < 0.01 vs. BCCAO + Pur (150 mg/kg/day); ^&^*P* < 0.05, ^&&^*P* < 0.01 vs. BCCAO + NGF (20 mg/kg/day).

Transferase dUTP nick end labeling staining detects apoptotic neurons. As depicted in Figure 4, BCCAO rats had increased amounts of TUNEL-positive neurons in the dorsal CA1 region in comparison with sham animals. Dorsal CA1 region nuclei in BCCAO rats had irregular shapes and showed degeneration. BCCAO animals administered Pur or NGF, specially Pur and NGF together, showed starkly reduced amounts of TUNEL-positive cells, indicating Pur and NGF protected neurons from apoptosis. Then, Pur’s effects on Bcl-2 and Bax expression (Figure 5) were examined. These two proteins were detected by Western blot and double immunofluorescence staining in the Sham, BCCAO, Sham + Pur (150 mg/kg/day), BCCAO + Pur (50 mg/kg/day), BCCAO + Pur (100 mg/kg/day), BCCAO + Pur (150 mg/kg/day), BCCAO + NGF (20 mg/kg/day), and BCCAO + NGF (20 mg/kg/day) + Pur (150 mg/kg/day) groups, respectively. Both western blot and double immunofluorescence staining demonstrated significantly downregulated Bcl2, starkly upregulated Bax and significantly elevated Bax/Bcl-2 ratio in the BCCAO group in comparison with the sham group. However, Pur treatment alleviated these changes dose-dependently. The BCCAO + NGF (20 mg/kg/day) + Pur (150 mg/kg/day) groups showed better improvement than the NGF (20 mg/kg/day) and Pur (150 mg/kg/day) monotherapy groups.

### Puerarin Suppresses Occlusion of Bilateral Common Carotid Arteries-Induced Oxidative Stress in the Dorsal CA1 Region of Hippocampus

**FIGURE 6:**
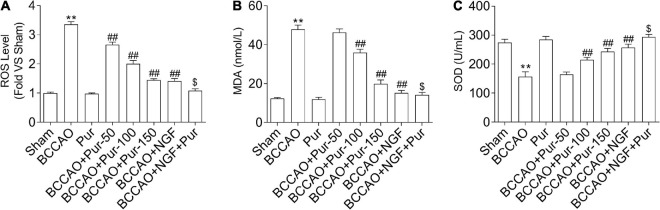
Effect of Pur on **(A)** ROS, **(B)** MDA, and **(C)** SOD levels in experimental rats. Results were mean ± SEM (*n* = 6). **P < 0.01 vs. Sham; ^##^*P* < 0.01 vs. BCCAO; ^$^*P* < 0.05 vs. BCCAO + Pur (150 mg/kg/day).

Reactive oxygen species production represents a by-product of mitochondrial oxidative phosphorylation, an important target of ROS-associated injury (Zhang et al., 2019b). It is known that ROS are biosynthesized early after BCCAO, compromising non-enzymatic and enzymatic antioxidant defense systems (Hara et al., 2002). Therefore, we firstly examined the homeostatic modulation (mainly refers to the change of ROS content) of mitochondrial ROS, which is involved in oxidative stress-related damage during BCCAO. The results demonstrated that ROS levels were remarkably elevated in BCCAO animals in comparison with sham rats (Figure 6A). Meanwhile, Pur dose-dependently alleviated BCCAO-associated ROS level increase (Figure 6A). In addition, MDA upregulation (Figure 6B) and SOD downregulation (Figure 6C) were observed after BCCAO. Pur alleviated the BCCAO-associated MDA upregulation and SOD downregulation (Figures 6B,C). Interestingly, NGF (20 mg/kg/day) + Pur (150 mg/kg/day) showed better effects than Pur (150 mg/kg/day) alone.

### Puerarin Induces Transient Receptor Potential Melastatin-Related 2 Inhibition and NR2A Activation in the Hippocampus of Occlusion of Bilateral Common Carotid Arteries Rats

**FIGURE 7:**
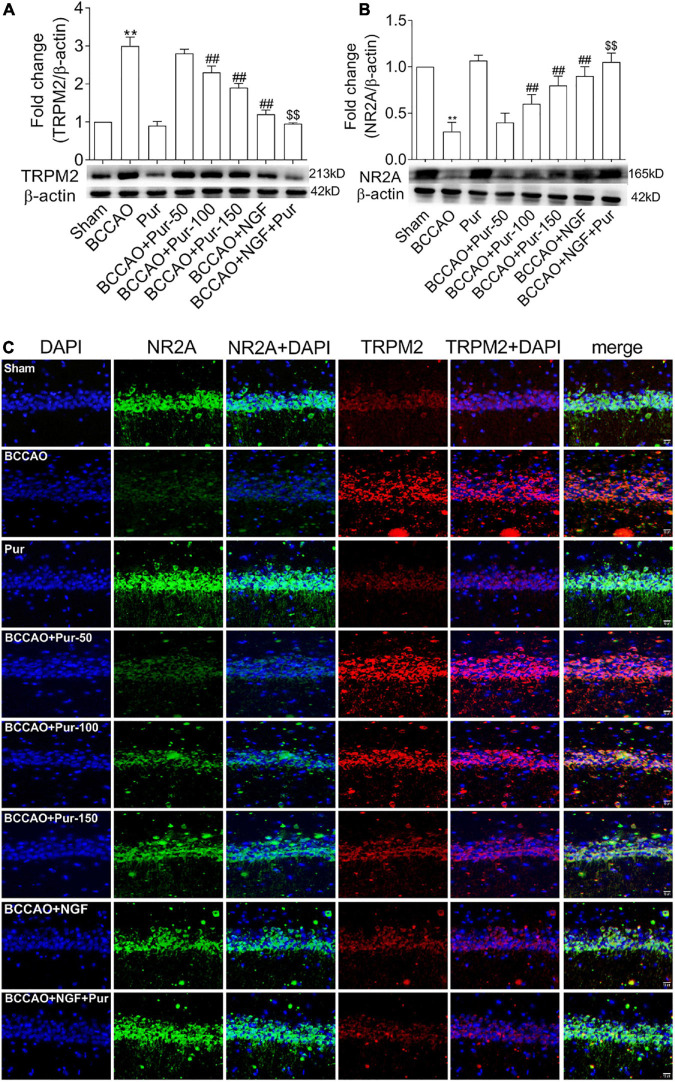
Expression and co-localization of NR2A and transient receptor potential melastatin-related 2 (TRPM2) in the rat hippocampus. **(A)** Protein amounts of TRPM2 in the hippocampal dorsal CA1 region of rats. **(B)** Protein amounts of NR2A in the hippocampal dorsal CA1 region of rats. **(C)** Effects of Pur on NR2A and TRPM2 co-localization in the hippocampal dorsal CA1 region, examined by immunofluorescence double staining. Representative micrographs are shown at ×200. Results were mean ± SEM (*n* = 6). **P < 0.01 vs. Sham; ^##^*P* < 0.01 vs. BCCAO; ^$$^*P* < 0.01 vs. BCCAO + Pur (150 mg/kg/day).

As Pur increased synaptic structural plasticity in BCCAO rats in this study, we hypothesized that the alleviated cognitive impairment and neuropathological alterations might be related to non-glutamatergic, Ca^2+^-dependent processes of ischemia-induced neuronal damage; meanwhile, TRPM2 was shown to mediate toxic Ca^2+^ influx (Hara et al., 2002; Aarts et al., 2003). In addition TRPM2 plays multiple physiological roles, contributing to inflammatory response and synaptic plasticity (Lange et al., 2009; Xie et al., 2011; Haraguchi et al., 2012). On the other hand, ischemia-induced neuronal death results from activated glutamate and non-glutamate-dependent channels, which promote cellular Ca^2+^ overload (Arundine and Tymianski, 2004; Aarts and Tymianski, 2005). Traditionally, glutamate receptor-related excitotoxicity results from over-activated *N*-methyl-D-aspartate glutamate receptors (NMDARs) (Besancon et al., 2008). Here we assessed whether Pur could trigger TRPM2 and NMDAR pathway effectors and downstream targets. To this end, NR2 activation and TRPM2 inhibition were assessed by double-immunofluorescence staining in neurons after BCCAO treatment. As shown in Figures 7A,B, treatment with Pur (100 or 150 mg/kg) obviously inhibited TRPM2 and activated NR2A compared with the BCCAO group, indicating a possible mechanism for Pur’s therapeutic effect. Then, TRPM2 and NR2A protein expression levels in the hippocampus were assessed by immunoblot. As shown in Figure 7C Pur treatment in BCCAO rats obviously reduced the protein expression of TRPM2 and increased NR2A expression. Notably, treatment with Pur (150 mg/kg) plus NGF (20 mg/kg/day) of BCCAO rats showed better effects than Pur (150 mg/kg) only.

## Discussion

Oxidative stress or high ROS amounts and calcium overload are critically involved in nerve cell apoptosis and vascular vasorelaxation dysfunction (Versari et al., 2009; Weseler and Bast, 2010; Gocmez et al., 2019), pathologically characteristic of BACCO-induced VD in rats. Puerarin (Pur), an isoflavone, is known to alleviate multiple cerebrovascular diseases. However, the mechanisms behind anti-apoptosis and cardiac/cerebrovascular protective effects of Pur in nerve and endothelial cells in BACCO-induced VD remain undefined. This study firstly demonstrated that Pur effectively inhibited nerve cell apoptosis and maintained endothelial cell function, which might be via suppressing oxidative stress and calcium overload. As shown above, Pur inhibited nerve cell apoptosis and maintained endothelial cell function by reversing oxidative stress *in vivo* (Figure 6). A parallel decrease in the levels of Bax and Bax/Bcl2 and an increase in endothelial cell function in BACCO-induced VD rats were observed (Figures 2, 5). Furthermore, we found that Pur could be utilized to treat VD, which might be through ROS-dependent TRPM2/NMDAR pathway suppression.

Puerarin presented remarkable effects in BACCO-induced VD rats. The initial MWM results showed that BCCAO rats were significantly deficient in learning and memory abilities at 24–28 days post-surgically. In the subsequent MWM, Pur-treated rats had markedly reduced total escape latency, while the BCCAO group showed the longest escape latency. The neuron is a key unit for the structural and functional basis of the central nerves system; neurons are distributed in the whole brain, especially in some unique areas such as the dorsal CA1 region of hippocampus that is associated with cognition, memory and learning (Du et al., 2017). H&E staining disclosed that rats after the BCCAO surgery had multiple neurons arranged sparsely and disorderly, unclear boundary between the cell contour and surrounding tissue and neuronal cell loss. Meanwhile, treatment with Pur reversed these histological alterations. Golgi-Cox staining clearly showed the morphological details of synapses, including the number of spines and the percentage of mushroom spines. Alterations in synaptic connections are crucial for learning and memory formation (Yang et al., 2009). Therefore, we examined synaptic changes in the dorsal CA1 region of hippocampus by Golgi-Cox staining. Pur obviously increased the number of spines and the percentage of mushroom spines. These findings revealed that Pur treatment improves cognitive function and ameliorates the morphological lesions in the dorsal CA1 region of hippocampus.

Apoptosis promotes cell loss featured by multiple neurological pathologies such as AD and VD (Favaloro et al., 2012). We here used TUNEL staining, double-immunofluorescence staining and immunoblot to examine Bax and Bcl-2 expression in the dorsal CA1 region of hippocampus. TUNEL staining showed induced cell apoptosis, alongside upregulated proapoptotic factors (e.g., Bax) and downregulated anti-apoptotic markers (e.g., Bcl-2) in VD model rats. These effects were reversed upon Pur administration. These findings suggest Pur ameliorates cognitive impairment by reducing hippocampal neuron apoptosis, the major contributor to memory decline in individuals with dementia.

Vascular dementia is often accompanied by endothelial dysfunction, and elevated oxidative stress is considered a potential mechanism leading to suppressed endothelium-dependent relaxation (Versari et al., 2009; Weseler and Bast, 2010; Gocmez et al., 2019). As ROS represent by-products of mitochondrial oxidative phosphorylation, this study assessed mitochondrial ROS levels, which were starkly increased in the BCCAO group in comparison with the sham group. Moreover, reduced SOD and increased MDA amounts were observed in BACCO rats in comparison with non-treated rats. However, after treatment with Pur or NGF, oxidative stress indexes were reversed. To examine Pur’s effect on endothelial dysfunction, endothelium-dependent (acetylcholine), and -independent (sodium nitroprusside) relaxations were assessed in thoracic aortic rings. Pur could alleviate vascular dysfunction in an endothelium-dependent manner. These findings revealed that Pur treatment decreases oxidative stress and ameliorates endothelial dysfunction.

Transient receptor potential melastatin-related 2 belongs to the TRPM subfamily, which is a part of the TRP superfamily (Venkatachalam and Montell, 2007). It was previously termed long TRPC2 (LTRP2) (Perraud et al., 2001; Sano et al., 2001). TRPM2 is a Ca^2+^-permeable channel (Perraud et al., 2001; Xia et al., 2008) effectively induced by ROS. TRPM2 is found in hippocampal, cortical and striatal neurons (Jiang et al., 2010). Consistent evidence indicates an important role for TRPM2 in delayed neuronal death and cognitive dysfunction (Verma et al., 2012; Shimizu et al., 2013; Gelderblom et al., 2014). Additionally, TRPM2 KO markedly improves neurological outcomes such as learning and memory following transient focal or global ischemic insult (Perraud et al., 2001; Ye et al., 2014), further indicating a critical role for TRPM2-assocated delayed neuronal death in cognitive dysfunction. Recently, pharmacological inhibition of TRPM2 channels was shown to obviously attenuate cognitive deficits in rodent models of diabetes (Thapak et al., 2020), global ischemia (Dietz et al., 2020), chronic cerebral hypoperfusion (Miyanohara et al., 2018), aging (Kakae et al., 2019), epilepsy (Zheng et al., 2020) and AD (Jiang et al., 2018). Based on these novel findings, it was hypothesized that TRPM2 could be targeted in a therapeutic strategy for alleviating brain injury and cognitive impairment associated with the above conditions. Therefore, we assumed that TRPM2 channels play an important role in cognitive dysfunction in VD rats, and its underlying mechanism need to be deduced.

The NMDAR pathway is very important in neuronal survival and death (Hardingham and Bading, 2003; Lai et al., 2014). Many molecules activate NMDAR signaling to prevent VD in rats (Zhang N. et al., 2015; Xing et al., 2016). NMDAR signaling is a representative pathway that affects the VD process (Meng et al., 2008; Liu et al., 2016). More specifically, NR2A pathways are neuroprotective, while NR2B signaling pathways induce neuronal death (Liu et al., 2007; Soriano et al., 2008; Terasaki et al., 2010; Alim et al., 2013). TRPM2^–/–^ animals show enhanced synaptic excitability in hippocampal neurons, which is suppressed by NR2A downregulation. Consistently, TRPM2^–/–^ selectively and simultaneously upregulates GluN2A and downregulates GluN2B in hippocampal neurons (Alim et al., 2013). Therefore, it was proposed that TRPM2 activation inhibits GluN2A-related survival signaling pathways and induces GluN2B-related signaling, causing neuronal death (Alim et al., 2013). For this reason the most probable mechanism of TRPM2-mediated neuronal death in VD may involve the NMDAR pathway. However, until now the function of TRPM2 conductance in ischemia-related Ca^2+^ influx has been hard to assess due to lack of specific regulators (Jiang et al., 2010). Based on these findings, it is crucial to develop and assess TRPM2 suppressors for VD treatment.

On the basis of the above results that Pur treatment improved cognitive and endothelial functions, we hypothesized that such improvements are associated with Pur’s modulation of ROS-dependent TRPM2/NMDAR signaling pathway. TRPM2 in neurons was strongly activated in rats with BCCAO; however, after Pur treatment, especially at 150 mg/kg, TRPM2 was significantly suppressive. At the same time, we focused on NR2A alteration both in normal, BCCAO and drug-treated rats. The results showed that NR2A was activated by Pur treatment. Therefore, TRPM2 might be required for GluN2A subunit downregulation and promoted neuron death. This phenomenon was in accord with a study assessing hippocampal samples from TRPM2^–^/^–^ animals revealing that TRPM2 deficiency results in neuroprotection by regulating GluN2A activity (Alim et al., 2013). NR2A accumulates at synapses and contributes to cell survival and synaptic excitation (Arundine and Tymianski, 2004; Aarts and Tymianski, 2005). Western blot indicated TRPM2 might be involved in regulating NR2A in the NMDAR pathway in VD formation, which was consistent with the above double-immunofluorescence staining data. Administration of Pur could restrain TRPM2 expression and upregulate NR2A in the NMDAR pathway, suggesting Pur to be an acute TRPM2 inhibitor in VD rats.

In our experiment, we chosen NGF to evaluate the therapeutic effect of Pur in treating the BACCO rats. The dysfunction of cholinergic neurons is related to cognitive defects (Davies and Maloney, 1976), and mature cholinergic neurons in the basal forebrain are highly dependent on NGF signals (Fischer et al., 1987). NGF was initially found to be a signal molecule in nerve cells, an endogenous molecule necessary for survival, differentiation, and maintenance of synaptic plasticity of cholinergic neurons (Winkler et al., 1998; Fiore et al., 2009). NGF can inhibit neuronal oxidative stress (Sun et al., 2017), prevent neuronal loss (Thoenen et al., 1987), stimulate neurite outgrowth (Ishitani et al., 2009), and improve memory impairment (Bar et al., 2003). Due to these effects, NGF is a potential therapeutic target for dementia (Choi et al., 2011). Consistently, our results showed that NGF could attenuate spatial cognitive impairment (MWM test), repair neuropathological changes (H&E staining), recover the synaptic structure (Golgi staining, NR2A expression), inhibit neuronal oxidative stress (ROS/SOD/MDA level) and nerve cells apoptosis (Tunnel staining, Bax/Bcl2 expression). Previous researches found that NGF Induces endothelial cell invasion and Cord Formation (Park et al., 2007), NGF functions as an indirect activator of angiogenesis by inducing specific molecules, such as vascular endothelial growth factor (VEGF) (Calza et al., 2001) and VEGF expression may possibly contribute in improving endothelium-dependent vasorelaxation and protecting hearts from I/R injury in SHR during late phase of whole body hypoxic preconditioning (Hong et al., 2019). Thus, NGF may have the similar endothelium-dependent vasodilatory to Pur. And in our study, we verified NGF remarkably improved acetylcholine-associated endothelium-dependent relaxation in BCCAO animals. More importantly, NGF combined with Pur had better effect on treating BACCO induced vascular cognitive impairment.

This study still had some limitations, which should be further investigated in the future. (1) As we only detected the level or the expression ROS, TRPM2 and NR2A, et al. The mechanism that Pur alleviates neuronal cell loss and vascular injury through the ROS-dependent TRPM2/NMDAR pathway is lack of evidence. The direct evidence between ROS dependent TRPM2 and the NMDA pathway should be further explored *in vitro*, and TRPM2^–/–^ mice might be helpful in verifying its exact target and providing more comprehensive evidence to reveal Pur’s effects in VD. (2) This study just assessed the levels of NR2A in the NMDAR pathway; more subunits and cognition-related proteins such as PSD-95, which may be involved in this process, should be evaluated in VD (Chung et al., 2004). (3) Vascular damage resulting in multiple alterations in both vascular and cerebral homeostasis may trigger the subsequent TRPM2 caspase cascade, and Pur may reverse this process in both outset and terminal links. These assumptions need more proof combining systemic and dynamic evidences to unfold TRPM2 modulation in VD and Pur’s therapeutic effects.

To conclude, Pur markedly improves cognitive impairment associated with BCCAO in rats. In this study, Pur overtly alleviated neuron injury, spine alterations and apoptosis. The process may involve TRPM2 suppression and induced NR2A in the NMDAR pathway leading to cell survival. Pur from traditional Chinese medicine is a promising TRPM2 modulator and treatment option for VD patients. It is presently unknown whether TRPM2 acutely impacts cognitive function in VD rats through the NMDAR pathway or other signaling cascades. Thus, the mechanisms by which TRPM2 modulates NMDARs and likely other channels deserve a comprehensive assessment to identify more potent therapeutic targets for VD.

## Data Availability Statement

The original contributions presented in the study are included in the article/Supplementary Material, further inquiries can be directed to the corresponding authors.

## Ethics Statement

The animal study was reviewed and approved by Xinxiang Medical University Veterinary Medicine Animal Care and Use Committee. Written informed consent was obtained from the owners for the participation of their animals in this study.

## Author Contributions

PL and YY designed the research study. TZ wrote the manuscript. GW and SW revised the manuscript. MZ, ZW, and YQ did the animal caring and surgery. ZW and YQ performed the MWM. NH and MZ did the HE, Golgi, and TUNEL staining. JX and PS did the double-immunofluorescence staining. TZ and YQ did the Western blots. MZ and NH did the Elisa assay. All authors were involved in drafting this work for important intellectual content, agreed to the version published, and are accountable for all aspects presented in this study.

## Conflict of Interest

The authors declare that the research was conducted in the absence of any commercial or financial relationships that could be construed as a potential conflict of interest.

## Publisher’s Note

All claims expressed in this article are solely those of the authors and do not necessarily represent those of their affiliated organizations, or those of the publisher, the editors and the reviewers. Any product that may be evaluated in this article, or claim that may be made by its manufacturer, is not guaranteed or endorsed by the publisher.
