# Supplementary material for: Puerarin Alleviates Vascular Cognitive Impairment in Vascular Dementia Rats
Source: Front Behav Neurosci. 2021 Oct 15;15:717008. doi: 10.3389/fnbeh.2021.717008 (PMC8554240; doi:10.3389/fnbeh.2021.717008)
Supplement: Supplementary file 1 [file Table_1.docx]

Supplement table 1. the (BCCAO-Sham)/BCCAO Value of each BCCAO rat

| Number | (BCCAO-Sham)/BCCAO Value(＞0.2) |
| --- | --- |
| BCCAO1 | 0.3288934 |
| BCCAO2 | 0.294181 |
| BCCAO3 | 0.2488532 |
| BCCAO4 | 0.2348131 |
| BCCAO5 | 0.2453917 |
| BCCAO6 | 0.2275943 |
| BCCAO7 | 0.2383721 |
| BCCAO8 | 0.2239336 |
| BCCAO9 | 0.3002137 |
| BCCAO10 | 0.3527668 |
| BCCAO11 | 0.3119748 |
| BCCAO12 | 0.2383721 |
| BCCAO13 | 0.2348131 |
| BCCAO14 | 0.2275943 |
| BCCAO15 | 0.294181 |
| BCCAO16 | 0.3316327 |
| BCCAO17 | 0.2590498 |
| BCCAO18 | 0.2786344 |
| BCCAO19 | 0.2312207 |
| BCCAO20 | 0.2418981 |
| BCCAO21 | 0.3031915 |
| BCCAO22 | 0.3261317 |
| BCCAO23 | 0.2786344 |
| BCCAO24 | 0.2656951 |
| BCCAO25 | 0.3423695 |
| BCCAO26 | 0.2722222 |
| BCCAO27 | 0.2786344 |
| BCCAO28 | 0.2911255 |
| BCCAO29 | 0.2972103 |
| BCCAO30 | 0.2590498 |
| BCCAO31 | 0.4045455 |
| BCCAO32 | 0.2556818 |
| BCCAO33 | 0.3397177 |
| BCCAO34 | 0.2754425 |
| BCCAO35 | 0.3061441 |
| BCCAO36 | 0.2590498 |
