# Supplementary figures and images for: Puerarin Alleviates Vascular Cognitive Impairment in Vascular Dementia Rats
Source: Front Behav Neurosci. 2021 Oct 15;15:717008. doi: 10.3389/fnbeh.2021.717008 (PMC8554240; doi:10.3389/fnbeh.2021.717008)

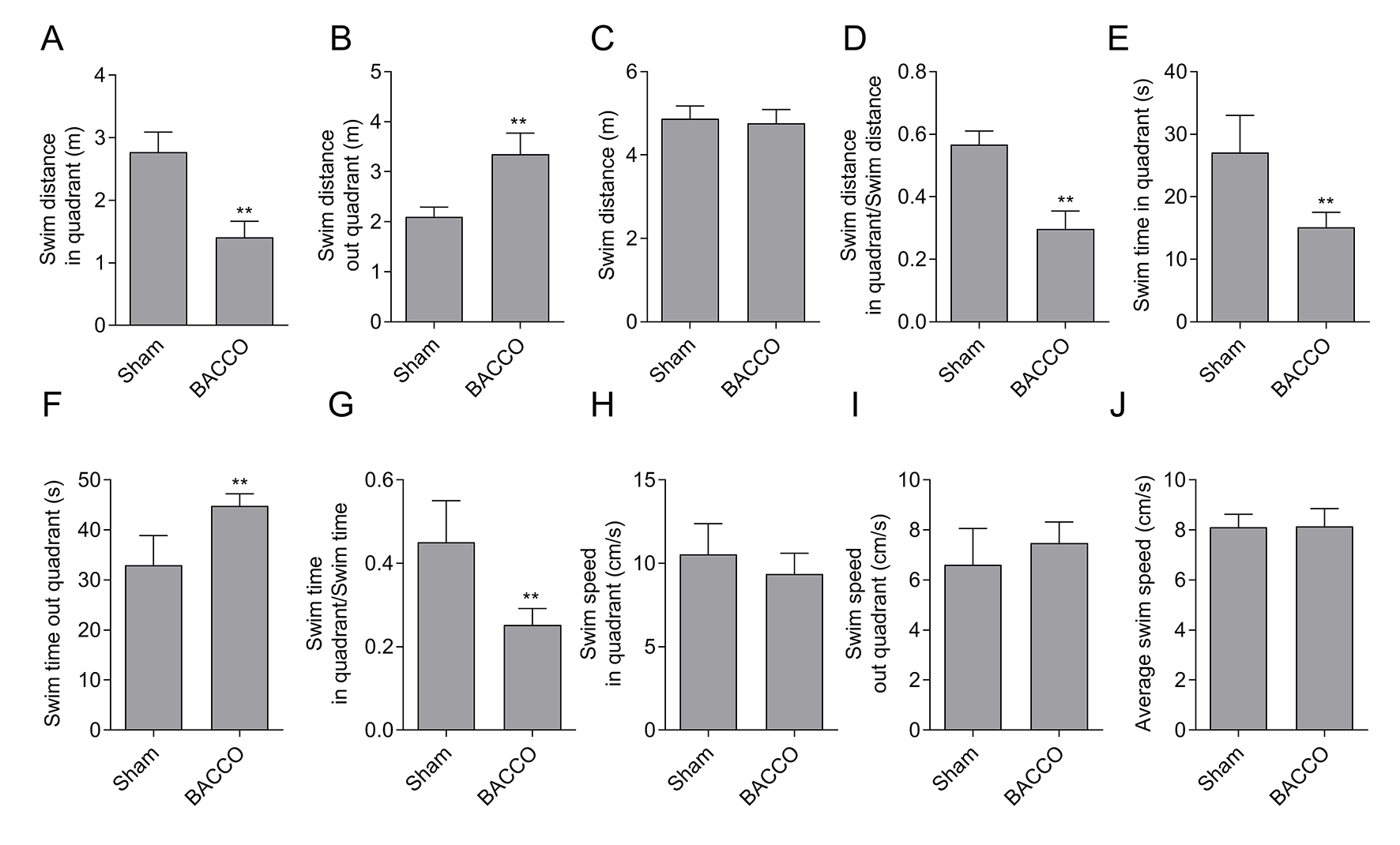

Supplement: Supplementary file 4 [file Image_1.TIF]
